# Human embryonic stem cell-derived mesenchymal cells preserve kidney function and extend lifespan in NZB/W F1 mouse model of lupus nephritis

**DOI:** 10.1038/srep17685

**Published:** 2015-12-02

**Authors:** Austin Thiel, Gregory Yavanian, Maria-Dorothea Nastke, Peter Morales, Nicholas A. Kouris, Erin A. Kimbrel, Robert Lanza

**Affiliations:** 1 Ocata Therapeutics, Marlborough, MA 01752.

## Abstract

Adult tissue-derived mesenchymal stromal cells (MSCs) are showing promise in clinical trials for systemic lupus erythematosus (SLE). However, the inability to manufacture large quantities of functional cells from a single donor as well as donor-dependent variability in quality limits their clinical utility. Human embryonic stem cell (hESC)-derived MSCs are an alternative to adult MSCs that can circumvent issues regarding scalability and consistent quality due to their derivation from a renewable starting material. Here, we show that hESC-MSCs prevent the progression of fatal lupus nephritis (LN) in NZB/W F1 (BWF1) mice. Treatment led to statistically significant reductions in proteinuria and serum creatinine and preserved renal architecture. Specifically, hESC-MSC treatment prevented disease-associated interstitial inflammation, protein cast deposition, and infiltration of CD3^+^ lymphocytes in the kidneys. This therapy also led to significant reductions in serum levels of tumor necrosis factor alpha (TNFα) and interleukin 6 (IL-6), two inflammatory cytokines associated with SLE. Mechanistically, *in vitro* data support these findings, as co-culture of hESC-MSCs with lipopolysaccharide (LPS)-stimulated BWF1 lymphocytes decreased lymphocyte secretion of TNFα and IL-6, and enhanced the percentage of putative regulatory T cells. This study represents an important step in the development of a commercially scalable and efficacious cell therapy for SLE/LN.

Systemic lupus erythematosus (SLE) is a debilitating multi-organ autoimmune disease that has no cure and limited treatment options^1^. SLE pathogenesis is complex and involves the loss of tolerance to nuclear self-antigens, including double-stranded DNA and chromatin, leading to immune-complex-mediated inflammation and tissue damage in affected organs^2^,^3^. Various immune cell populations, including B^4^,^5^ and T cells^6^,^7^, macrophages^8^, natural killer cells^9^, dendritic cells^10^,^11^ and their secreted cytokines^12^,^13^, contribute to this pathogenic process, making the treatment of this multi-faceted disease particularly challenging.

Current therapies, e.g. antimalarials such as hydroxychloroquine, non-steroidal anti-inflammatories, glucocorticoids, and for the most severe cases, general immunosuppressants such as mycophenolate mofetil are not curative and elicit adverse side-effects, particularly with long-term use^1^,^14^. In addition, certain patient groups, such as those with kidney involvement- i.e., lupus nephritis (LN), are often refractory to such treatments^15^, highlighting the need to develop more effective therapies. Considerable time and effort has been spent in developing targeted therapies to fight SLE, yet only one therapy, belimumab (Benlysta), a monoclonal antibody targeting B cell-activating factor, or BAFF, has been approved for the treatment of SLE in the last half-century^16^. Unfortunately, more than 40% of belimumab-treated SLE patients failed to display a clinical response in Phase III trials^17^,^18^.

The lack of success in developing safe and effective SLE therapies based on small molecules or biologics has led investigators to test cell-based therapies, such as mesenchymal stem/stromal cells (MSCs), which can be isolated from various tissues, including bone marrow, umbilical cord, placenta, and adipose tissue^19^. Evidence shows that MSCs home to sites of inflammation where they inhibit immune and inflammatory responses by influencing the behavior of local innate and adaptive immune cells (reviewed in^20^). MSCs may be advantageous over other SLE therapies due to their ability to target multiple components of an autoreactive immune system while allowing recipients to retain a functional immune response against infectious agents and eliciting few side-effects^21^,^22^.

The precedent for using MSCs in SLE has been set by several small clinical trials which have shown that MSCs are safe and efficacious in human patients refractory to standard treatment or with severe disease^23^,^24^,^25^,^26^. BM and UC-MSC single dose infusions led to a decrease in SLEDAI (systemic lupus erythematosus disease activity index) scores; notably proteinuria, creatinine and blood urea nitrogen levels were all reduced. Patients were followed for up to 4 years, showing strong rates of survival and remission^27^. These studies hint at the promise of using mesenchymal cells to treat SLE. However, these trials were not randomized and larger studies will be necessary in order to fully evaluate the therapeutic value of MSCs for SLE therapy. With larger studies, MSC scalability and preservation of therapeutic functionality will become problematic for cells derived from adult tissues.

Evidence indicates that both the age of the donated tissue and extended *in vitro* culture can negatively affect MSC quality and thus their therapeutic effect. MSCs derived from older donors (e.g., adult bone marrow) lose functionality sooner than those derived from young (e.g., fetal, placental, or embryonic) donor tissue^28^,^29^,^30^. Likewise, extended *in vitro* culture impairs MSC homing and immunomodulatory ability^31^,^32^,^33^,^34^. MSCs that have undergone only limited *in vitro* expansion appear to perform better in clinical trials than those which have been extensively expanded^35^. MSCs derived from adult tissues are not replenishable, and if not extensively expanded, must constantly be derived from different donors, contributing to inconsistencies in their clinical performance. Given these issues, a renewable source of MSCs from young tissue would provide a more potent, consistent, and reliable MSC therapeutic product, one that would allow large scale manufacturing without the need for extended *in vitro* culture, thus preserving their therapeutic efficacy.

Recently, we have demonstrated the ability to produce cells with many of the same characteristics (e.g., cell surface markers, differentiation capacity, secreted cytokines, immunomodulatory properties) as MSCs but from pluripotent hESCs, a renewable and young cell source^36^. Our previous work showed that hESC-MSCs are more effective than human bone marrow-derived MSCs in reducing paralysis in an experimental autoimmune encephalomyelitis (EAE) model for multiple sclerosis^37^. In proof of concept experiments, our hESC-MSCs were also able to reduce immune cell infiltrate and preserve retinal architecture in an experimental autoimmune uveitis (EAU) model and increase the survival of lupus-prone New Zealand Black (NZB) × New Zealand white (NZW) mice F1 generation (or BWF1) mice^36^. BWF1 mice are a classic, well-studied spontaneous model for SLE with a reduced lifespan due to lupus-associated, immune complex-mediated glomerulonephritis. The aim of the current study was to determine how hESC-MSCs affect lupus progression, and in particular, the development of lupus-associated glomerulonephritis. Here, we monitor BWF1 kidney function as a tractable read-out for autoimmune disease progression and use the BWF1 model to test the therapeutic effects of hESC-MSCs, a renewable cellular therapy, for SLE/LN.

## Results

### Characteristics of hESC-MSCs

**Figure 1:**
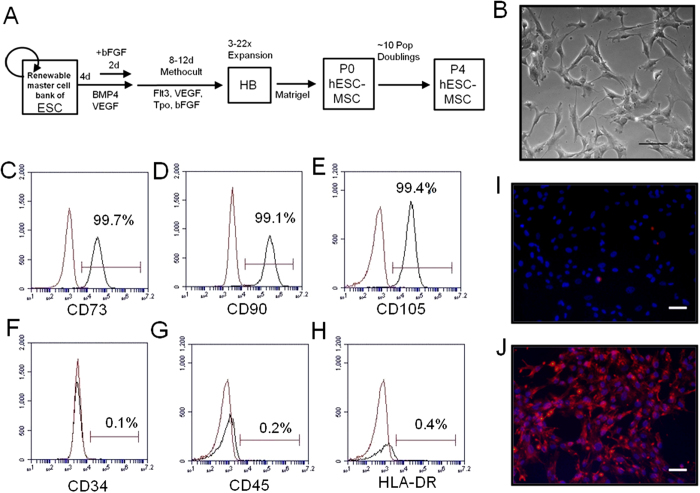
hESC-MSC derivation and phenotype. (**A**) Schematic outline of the process of differentiation from hESCs to hESC-MSCs. (**B**) hESC-MSC morphology under bright field imaging (Scale bar 100 μm). (**C**–**H**) Flow cytometry analysis of markers positive (**C**–**E**) and negative (**F**–**H**) for hESC-MSCs. Red histogram represents unstained populations. Average of two independent experiments. (**I**,**J**) Isotype control (**I**) and HLA-G (**J**) staining of hESC-MSCs (red). Cells were counterstained for DAPI (blue) (Scale bar 50 μm). Staining was performed twice, producing comparable results.

We derived hESC-MSCs from MA09 hESCs as previously described^36^. This process involved differentiating hESCs into embryoid bodies (EBs) for 4 days, followed by trypsinization into a single cell suspension and culture in a semi-solid cytokine-rich methylcellulose-based medium for 8–12 days to produce hemangioblasts, which exhibit between 3- and 22-fold expansion from the EB stage. We harvested, rinsed and transferred cells to matrigel-coated plates in hESC-MSC media, and termed the adherent cell population passage (P) 0 hESC-MSCs. (Figure 1A).

hESC-MSCs were expanded to P4 for use in the lupus mouse model over the course of ~20–22 days. The cells undergo approximately 10 population doublings during this time period^36^. Importantly, using a replenishable master cell bank of hESCs as starting material allows this process to be performed iteratively, thus providing a way to accumulate commercial scale quantities of genotypically identical MSCs that have never been cultured or expanded beyond 20–22 days. Our hESC-MSCs exhibit a fibroblast-like morphology (Fig. 1B) and express many of the same cell surface markers as MSCs derived from primary tissue sources. This includes a CD73^+^/CD90^+^/CD105^+^ and CD34^−^/CD45^−^/HLA-DR^−^ signature (Fig. 1C–H) but also higher expression of CD10 and CD24 and lower expression of Stro-1 compared to primary tissue MSCs, as we have previously reported^36^. In addition, we performed immunofluorescence for the non-classical HLA class I molecule, HLA-G, which is involved in the inhibition of immune cell function and in allowing allogeneic MSCs to evade immune cell-mediated clearance^38^. While an isotype control antibody gave no background staining (Fig. 1I), an anti HLA-G antibody showed hESC-MSCs stain positively for this marker (Fig. 1J).

### hESC-MSC administration delays LN disease progression in lupus-prone mice

**Figure 2:**
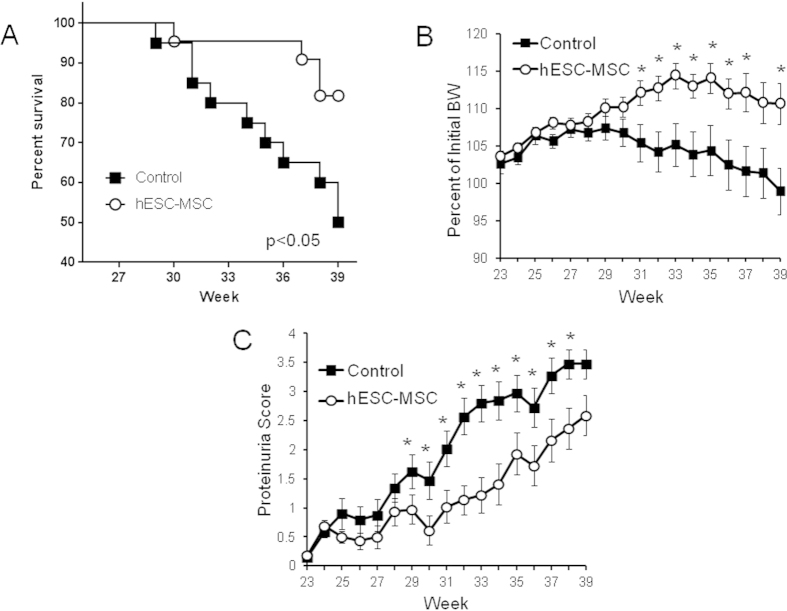
hESC-MSC treatment prolongs the survival of BWF1 mice. (**A**) Kaplan-Meier survival curve through week 39 for control (n = 20) and hESC-MSC (n = 22) treated mice (p < 0.05, log-rank). (**B**) Percent body weight change compared with week 20, before disease onset, for control and hESC-MSC-treated mice (n = 20 control, n = 22 hESC-MSC) (*p < 0.05, Mann-Whitney). (**C**) Average proteinuria score of control (n = 20, black boxes) or hESC-MSC-treated (n = 22, white circles) BWF1 mice (*p < 0.05, Mann-Whitney).

To determine whether our hESC-MSCs can influence lupus disease progression, we utilized BWF1 mice, a well-characterized strain which spontaneously develops an SLE-like autoimmune disorder and glomerulonephritis, the main cause for their reduced lifespan of 7 to 11 months (28–44 weeks)^39^,^40^. We intravenously injected BWF1 mice with 5 × 10^5^ hESC-MSCs, as we had previously used this amount in our pilot studies^36^, and injected them in a bi-weekly fashion from 23–33 weeks of age. hESC-MSC treatment significantly prolonged survival in these mice; 50% of controls either died or reached the criteria for euthanasia by week 39, whereas only 20% of hESC-MSC-injected mice did so by this time (Fig. 2A), thus confirming results of our pilot studies. To determine the beneficial effects of hESC-MSC treatment, we monitored body weight as a measure of animal health. hESC-MSC-treated mice maintained a higher percentage of their initial body weight, on average, compared to controls, suggesting that mice in the hESC-MSC cohort remained healthy for a longer period of time (Fig. 2B). We also monitored kidney function by estimating proteinuria (protein in the urine) once per week for 16 weeks (4 months). Over the course of several weeks, we observed the characteristic rise in proteinuria levels for BWF1 mice, indicative of lupus-associated glomerulonephritic disease. However, hESC-MSC-treated mice displayed significantly reduced proteinuria scores from 29–38 weeks of age compared to vehicle-treated controls (Fig. 2C). Together, these data demonstrate that hESC-MSC treatment can prolong survival in a lupus-prone mouse model and delay SLE disease progression.

### Lupus-prone mice treated with hESC-MSCs have reduced disease severity

**Figure 3:**
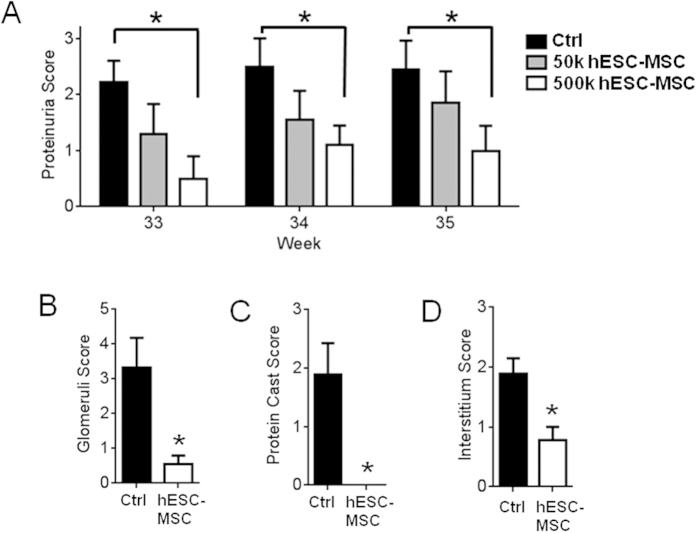
hESC-MSCs reduce proteinuria and kidney damage scores in BWF1 mice. (**A**) Analysis of average proteinuria score for weeks 33–35 in an age-matched subset of BWF1 lupus prone mice, treated with a low dose of hESC-MSCs (50k cells, n = 10, gray columns) or high dose (500k, n = 10, white), compared to controls (n = 9, black) (*p < 0.05, Mann-Whitney). (**B**–**D**) Average renal histopathology scores for glomeruli (**B**), protein cast formation (**C**), and interstitial inflammation (**D**). n = 9 control and n = 9 hESC-MSC (500k) (p < 0.05, Mann-Whitney).

In order to optimize hESC-MSC dosage, we treated BWF1 mice weekly from 24 to 26 weeks of age with either 5 × 10^4^ or 5 × 10^5^ cells, and monitored disease progression by measuring proteinuria. Average proteinuria scores were reduced compared to vehicle-injected controls at 33–35 weeks of age for both hESC-MSC treatment groups, although only the 5 × 10^5^ group was statistically significant (Fig. 3A). Given that the 5 × 10^5^ group had statistically significant differences in proteinuria scores from control, we sacrificed mice in this cohort at week 35 to perform age-matched kidney analysis of controls versus hESC-MSC-treated animals. Hematoxylin and eosin (H&E)- and periodic acid-Schiss (PAS)- stained kidney sections were analyzed by a blinded veterinary histopathologist and scored in three categories for evidence of disease. The average pathological scores for hESC-MSC-treated mice were significantly lower in each of the three scoring categories compared to vehicle-injected controls (Fig. 3B–D). Table 1 lists the individual histopathology scores for vehicle-treated controls and the 5 × 10^5^ treatment group along with their week 35 proteinuria scores. In general, high proteinuria scores correlated with high histopathology scores.

**Figure 4:**
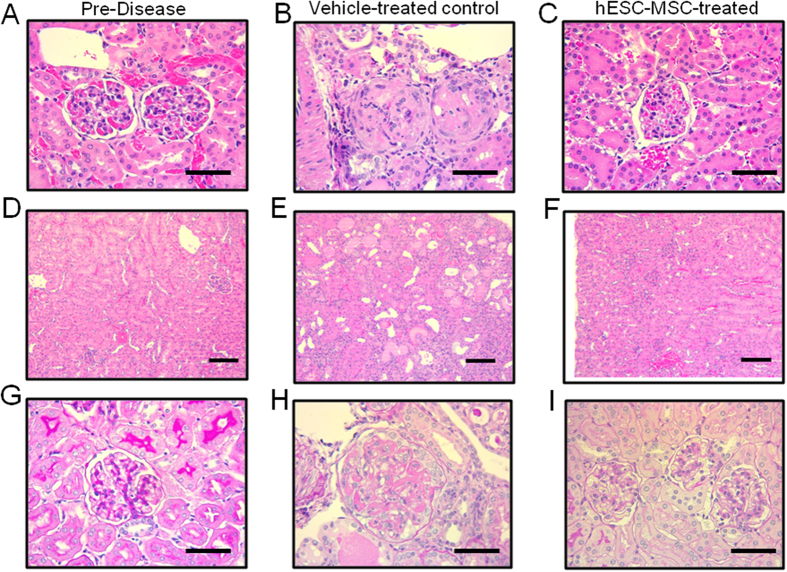
hESC-MSC treatment improves renal pathology in BWF1 mice. (**A–F**) H&E stained kidney sections from pre-disease, 15 week old mice (**A**,**D**), vehicle-treated 35 wk old controls (**B**,**E**) and hESC-MSC-treated (500K dose) 35 wk old mice (**C**,**F**). Scale bars (**A**–**C**) 50 μm, (**D**–**F**) 100 μm (**G**–**I**) PAS stained kidney sections from pre-disease, 15 week old mice in (**G**), vehicle-treated 35 wk old controls in (**H**) and hESC-MSC-treated (500K dose) 35 wk old mice in (**I**). Scale bars 50 μm. Results from each panel are representative of stains performed on tissue from three animals in each group.

**Figure 5:**
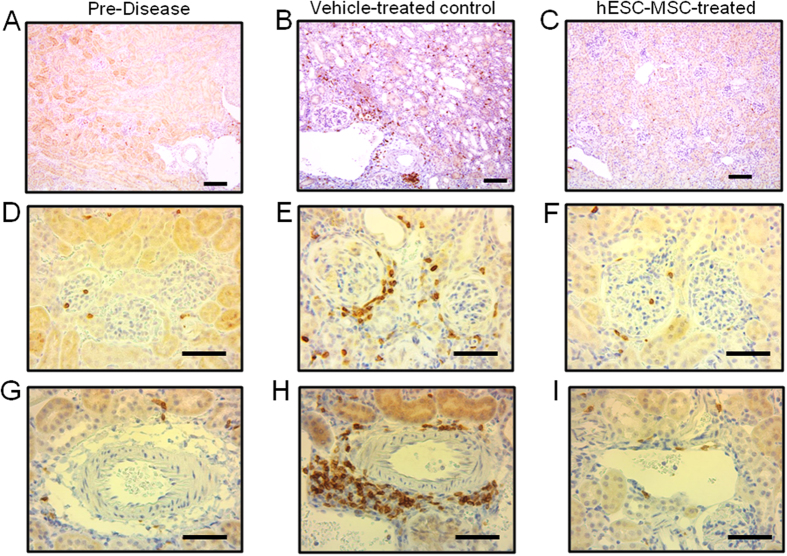
hESC-MSC treatment reduces renal immune cell infiltration in BWF1 mice. Immunohistochemical CD3-stained kidney sections (brown cells) from pre-disease, 15 week old mice (**A**,**D**,**G**), vehicle-treated 35 wk old controls (**B**,**E**,**H**) and hESC-MSC-treated (500 K dose) 35 wk old mice (**C**,**F**,**I**). Scale bars 100 μm (**A**–**C**), 50 μm (**D**–**I**). Results from each panel are representative of stains performed on tissue from three animals in each group.

Kidneys from healthy, pre-disease 15 week old BWF1 mice contained small, well-structured glomeruli, and no protein cast formation (Fig. 4A,D,G). In contrast, kidneys isolated from more than half of the vehicle-treated control mice contained abundant mesangial deposits and cellular crescents at 35 weeks of age (Fig. 4B,H). Protein cast formation was also evident throughout the kidneys of 35 week-old vehicle-treated controls (Fig. 4E). In comparison, glomeruli from 35 week old (age-matched) hESC-MSC-treated mice showed minor, if any, pathological features, and looked similar to young pre-disease mice (Fig. 4C,F,I). In addition, kidneys from hESC-MSC-treated mice had markedly less CD3^+^ cell infiltration than vehicle-treated mice throughout the kidney (Fig. 5B,C), as shown in both the periglomerular (Fig. 5E,F) and perivascular (Fig. 5H,I) regions. Kidneys from hESC-MSC-treated mice more closely resembled pre-disease controls (Fig. 5A,D,G) with only scant CD3^+^ staining. We also performed immunostaining to examine kidney IgG accumulation. While the intensity of staining was variable, 3 out of the 9 samples from vehicle-treated mice stained with a greater intensity than any of the 9 samples from hESC-MSC-treated mice (Supplementary Fig. S1). Overall, we observed a slightly higher average staining intensity for controls than hESC-MSC-treated mice (Supplementary Fig. S1). Collectively, these results support the finding that hESC-MSC treatment prevents or slows lupus-associated glomerular disease in BWF1 mice.

### hESC-MSC treatment reduces signs of inflammation in the serum of BWF1 mice

**Figure 6:**
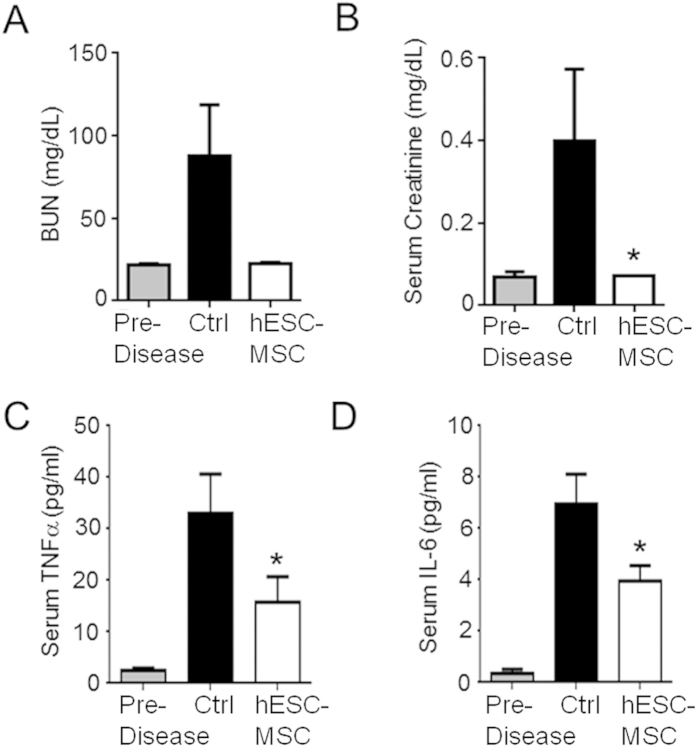
hESC-MSC treated animals display reduced serum cytokines and metabolites. (**A**) BUN levels in pre-disease (15 weeks of age, n = 5) (gray column), week 35 control (n = 9, black), and 500k hESC-MSC-treated (n = 9, white) BWF1 mice. (**B**) Serum creatinine levels in pre-disease (15 weeks of age, n = 5) (gray column), week 35 control (n = 9, black), and 500 k hESC-MSC-treated (n = 9, white) BWF1 mice. (*p < 0.05, ctrl vs. hESC-MSC, Mann-Whitney). (**C**,**D**) Cytokine levels of TNFα (**C**) and IL-6 (**D**) in pre-disease (15 weeks of age, n = 8), control (n = 12), and hESC-MSC-treated (n = 10) BWF1 mice (**p < 0.0005 for pre-disease vs ctrl; *p < 0.05 for ctrl vs. hESC-MSC, Mann-Whitney).

The metabolites BUN and creatinine accumulate in the serum as a result of kidney dysfunction in both SLE/LN patients and BWF1 mice. We observed a reduction in these serum markers in hESC-MSC-treated BWF1 mice at 35 weeks of age (Fig. 6A,B) compared with vehicle-treated controls. We also observed that serum levels of the inflammatory cytokines TNFα and IL-6 increase with age-associated disease progression (Fig. 6C,D, first and second bars). hESC-MSC treatment afforded a statistically significant reduction in these levels as compared to age-matched vehicle-treated controls (Fig. 6C,D, second and third bars) yet had little to no effect on serum levels of IL-2, IL-4, IL-10, IL-17, or IFNγ (data not shown). It should be noted that during our analysis of age-matched 35 week old BWF1 mice, we saw no changes in the levels of anti-double stranded DNA (Supplementary Fig. S2) or anti-nuclear antigen antibodies (data not shown). It is possible that hESC-MSC treatment afforded a transient decline in these antibodies earlier in the disease process, yet the effects were no longer apparent by the time of our serum analysis at week 35.

### hESC-MSCs directly affect BWF1 lymphocyte cytokine secretion and T cell populations

**Figure 7:**
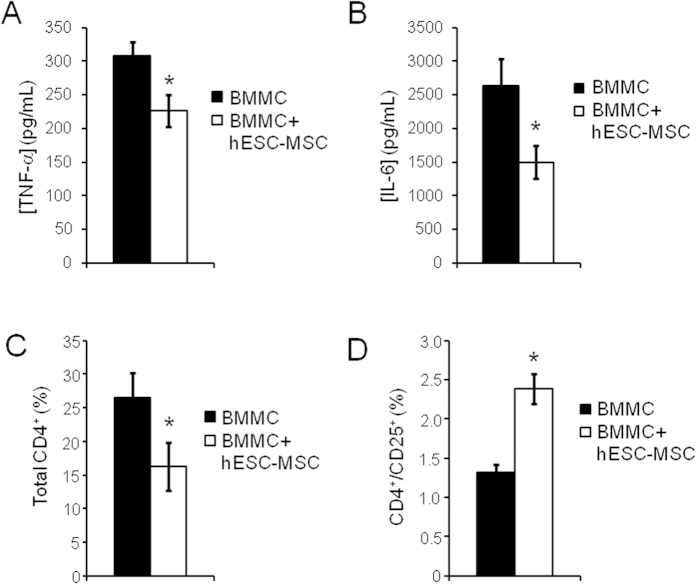
Co-culture of BMMCs from 35-week old BWF1 mice with hESC-MSCs reduces inflammatory cytokines and alters the CD4^+^ T-cell population. (**A**,**B**) Concentration of TNF-α (**A**) and IL-6 (**B**) in the supernatant of BMMC cultures with (n = 9) or without (n = 9) co-cultured hESC-MSCs. Cells were stimulated with LPS for 24 hrs (*p < 0.05, Mann-Whitney). (**C**,**D**) The percentage of total CD4^+^ (**C**) and CD4^+^/CD25^+^ (**D**) BMMCs either cultured alone (n = 8) or in combination with hESC-MSCs (n = 8) for 5 days with LPS stimulation for the final 72 hrs (*p < 0.05, Mann-Whitney).

To further investigate the mechanism by which hESC-MSCs delay SLE progression, we examined the direct effects of hESC-MSCs on BWF1 lymphocytes. We cultured bone marrow mononuclear cells (BMMCs) isolated from 35 week old BWF1 mice (n = 8, 4 with high (≥1000 mg/dL) proteinuria and 4 with low (trace amounts) proteinuria, either alone or in a co-culture with hESC-MSCs. The levels of cytokines secreted in the absence of lipopolysaccharide (LPS) stimulation were negligible (data not shown). However, 24 hours of LPS stimulation resulted in measurable amounts of various cytokines, most prominent of which were TNFα and IL-6. Consistent with our *in vivo* data on serum levels (Fig. 6C,D), both TNF-α and IL-6 levels were significantly reduced in the hESC-MSC co-cultures as compared to LPS-stimulated BMMCs alone (Fig. 7A,B). Other cytokines analyzed, IL-2, IL-4, IL-10, IL-17, and IFNγ were not significantly affected by hESC-MSC co-culture. There was also no difference in cytokine secretion by lymphocytes derived from low vs high proteinuria mice (data not shown).

In addition, we examined the effect of hESC-MSC co-culture on the T-cell population within BMMCs. We incubated BMMCs from 35-wk old BWF1 mice with or without hESC-MSC co-culture for 5 days, adding LPS for the last 3 days, and found that hESC-MSC co-culture reduced the total percentage of CD4^+^ cells (Fig. 7C). Surprisingly, however, the percentage of CD4^+^/CD25^+^ cells, a population that is enriched for regulatory T cells (Tregs), was significantly increased (Fig. 7D) in the hESC-MSC co-cultured samples as compared to BMMC alone. The absolute number of CD4^+^ and CD4^+^/CD25^+^ cells in culture followed the same pattern as our results for percentage changes (data not shown). This hESC-MSC-mediated effect occurred with lymphocytes isolated from both low and high proteinuria mice, which are pooled together in the graph.

## Discussion

Here, we show that hESC-MSCs, which are similar to adult tissue-derived MSCs but derived from a single and replenishable hESC source, provide a therapeutic effect in the BWF1 spontaneous mouse model for SLE. These results confirm our previous observation that hESC-MSC treatment prolongs the survival of these lupus-prone mice^36^, yet further shows that hESC-MSCs specifically slow the progression of glomerulonephritis and preserve kidney function in a statistically significant manner. Mechanistically, this effect may occur through an hESC-MSC-mediated reduction in inflammatory cytokine levels and enhancement of regulatory T cell populations. These results suggest that hESC-MSCs, a commercially scalable and potent alternative to adult MSCs, may be a suitable immunomodulatory therapy for the treatment of SLE/LN.

In our study, hESC-MSC treatment led to the preservation of kidney function as detected by a statistically significant reduction in proteinuria and serum creatinine levels, as well as a lower trend in BUN levels compared to non-treated controls. Renal architecture was significantly preserved as hESC-MSC treatment prevented age-associated mesangioproliferation, crescent formation, interstitial inflammation and protein cast formation that occurs in BWF1 kidneys as LN progresses. In addition, CD3-immune cell infiltration in the periglomerular and perivascular spaces of the kidneys from hESC-MSC treated mice was greatly reduced compared to that found in age-matched vehicle-treated controls, more closely resembling that found in young, pre-diseased animals. These observations are consistent with other reports which show that MSCs derived from murine BM^41^,^42^, human BM^43^, human adipose tissue^44^, and human umbilical cord^45^ delay the onset of lupus symptoms, with reduced proteinuria and improved kidney histology in the BWF1 and/or MRL/lpr lupus-prone strains. Yet, our findings are in contrast to a handful of reports which suggest that MSCs are not therapeutically effective in SLE models. In one such study, allogeneic BM-MSCs derived from healthy mice did not afford improvements in proteinuria or survival, despite improvements in renal pathology^46^. This study may have shown limited efficacy due to the use of high passage number MSCs (P20-25). Another study showed that BM-MSC administration did not improve kidney function or pathology and actually exacerbated disease^47^. This study compared one dose of treatment before (week 21) or after (week 32) disease onset. It is possible that these cells were not administered in the correct therapeutic window or with the dosage needed to impart therapeutically useful effects. Interestingly, in yet another study, autologous MSCs derived from 26–27 week old (post-disease onset) BWF1 mice were found to be less effective in slowing disease progression than those from B6 allogeneic mice or young (5–6 week old, pre-disease onset) BWF1 or MRL/lpr mice, suggesting an intrinsic defect in MSCs from mice with active disease^41^. This finding mirrors the situation in humans whereby autologous MSCs derived from SLE patients were found to be defective, showing increased senescence and apoptosis^48^, altered gene expression profiles in pathways governing cell cycle and cell adhesion^49^, and impaired migratory capacity^50^ as compared to MSCs derived from non-diseased controls. These studies underscore the finding that the therapeutic properties of MSCs vary with donor age or disease status and that extended *in vitro* culture compromises their functionality. The inability of certain MSC preparations, including autologous SLE patient-derived MSCs, to fight lupus highlights the need for a more reliable and replenishable therapeutic MSC product, such as hESC-MSCs.

In this study, hESC-MSCs were found to reduce levels of the inflammatory, potentially disease-contributing cytokines, TNF-α and IL-6. While serum levels of these cytokines increased as disease progressed, hESC-MSC treatment led to a statistically significant reduction in both factors. These results are further supported by *in vitro* data which showed that co-culture with hESC-MSCs caused statistically significant reductions in IL-6 and TNF-α secretion from LPS-stimulated BWF1 lymphocytes. In previous studies, anti-IL-6 antibody treatment was found to prevent severe LN in the BWF1 model^51^ while recombinant IL-6 treatment was found to accelerate disease^52^, demonstrating the SLE-promoting role of IL-6. Anti-IL-6 therapies are currently being tested in clinical trials and recent results from a Phase II study show that treatment with a IL-6 monoclonal antibody, PF-04236921 can reduce the frequency of SLE flares, particularly in patients with higher disease activity scores^53^. Therefore, the ability of hESC-MSCs to reduce IL-6 levels likely contributes to its effectiveness in delaying LN symptoms in BWF1 mice. Several studies indicate that SLE patients have elevated levels of circulating TNF-α, yet evidence suggests that directly blocking TNF-α may exacerbate auto-antibody production and symptoms of lupus in some patients^54^,^55^. TNF-α may have both disease-contributing and disease-fighting properties due to its divergent roles in inflammation and immune cell differentiation, proliferation, and apoptosis^56^. Therefore it remains to be determined if *indirectly* altering TNF-α levels, as hESC-MSCs have done in this study, may be beneficial for SLE patients and/or if the effect on TNFα contributes to the overall hESC-MSC therapeutic effect.

Another potential mechanism by which hESC-MSCs elicit a therapeutic effect is by increasing the population of regulatory T cells. A role for Tregs in the suppression of lupus has been previously demonstrated in the BWF1 mouse model^57^ and suggested in SLE patients. Co-culture of hESC-MSCs with LPS-stimulated lymphocytes caused a reduction in the overall CD4^+^ T cell pool. Interestingly, while the total percentage of CD4^+^ T cells decreased, there was a statistically significant increase in the percentage of CD4^+^/CD25^+^ double positive cells, a population enriched for regulatory T cells. Ongoing work is aimed at characterizing the expression of other regulatory T cell markers such as FoxP3 and/or CD62L in this putative Treg population and determining if adoptive transfer of a CD4^+^ T cell pool from hESC-MSC-treated animals may protect or alter the time course for disease progression in naïve lupus-prone mice.

The current study shows, for the first time, that hESC-derived MSCs can specifically alter lupus-associated glomerulonephritis and may serve as an alternative to primary tissue-derived MSCs. One of the main advantages of sourcing hESC-MSCs from a pluripotent stem cell source is that a virtually unlimited number of early passage (ie, minimally expanded) therapeutic cells can be generated simply by expanding the starting pool of hESCs. This allows hESC-MSCs to avoid issues that plague adult tissue-derived MSCs, ie, the aforementioned expansion-associated loss in potency and donor-dependent variation in quality. Of note however, safety considerations for a cell therapy derived from hESCs will be more stringent than for adult-derived cells due to the risk of pluripotent stem cell contamination in the final cell preparation. We have previously shown that our hESC-MSCs do not form teratomas in immunocompromised animals, an acid test for the presence of pluripotent stem cells^37^. In addition, hESC-derived therapies for other indications are being tested clinically and thus far have shown excellent safety profiles^58^, suggesting that safeguards can be put into place to mitigate the risks involved with the use of hESC-derived MSCs.

## Methods

### Derivation of hESC-MSCs using hemangio-derived mesenchymal cell (HMC^TM^) technology

MA09 hESCs, derived as previously described^59^, were cultured on irradiated mouse embryonic fibroblasts (Global Stem, Inc, Rockville MD), and maintained in Primate ES Cell Medium (Reprocell, Kanagawa, Japan) with 10 ng/ml basic fibroblast growth factor (bFGF, Peprotech). Cells were passaged with Dissociation Solution (Reprocell). hESC differentiation into MSCs was performed as previously described^36^ using our proprietary HMC^TM^ technology. Briefly, hESCs were subjected to 4 days of embryoid body (EB) differentiation followed by replating a single cell suspension in H4536 methylcellulose (Stem Cell Technologies, Vancouver, Canada) supplemented with FLT3 ligand, vascular endothelial growth factor (VEGF), thrombopoietin (TPO), and bFGF. After 10–12 days, small, light-refractive grape-like clusters of cells, called hemangioblasts, were abundant. Cells were collected^60^, rinsed, and plated on Matrigel (BD Biosciences, San Jose, CA) -coated tissue culture plates in hESC-MSC media (defined below). Adherent cells became passage (p)0 hESC-MSCs and were allowed to expand for 6–8 days prior to passaging. Adiopogenic, Osteogenic, and Chondrogenic differentiation of hESC-MSCs has been demonstrated previously^36^.

### hESC-MSC Culture and Flow Cytometry

hESC-MSCs were grown in MSC Medium (α-MEM, 20% Hyclone heat inactivated fetal calf serum; Thermo-Fisher, Waltham, MA; non-essential amino acids, P/S, glutamax; Life Technologies). For the first 3 passages, Matrigel-coated plates were used. Cells were subsequently plated directly onto tissue culture treated flasks. hESC-MSCs were passaged using 0.05% trypsin-EDTA, or TrypLE (Life Technologies). Cells were split when 70–80% confluent, and re-seeded at 4,500–5,000 cells/cm^2^. Flow cytometry was performed on a BD Accuri C6 flow cytometer (Becton Dickinson), using hESC-MSCs at passage 4–5. The following antibodies were used to analyze cell surface markers: CD73-PE, CD45-APC, CD34-FITC (BD); CD105-APC, CD90-FITC, CD24-APC, CD10-FITC, CD13-APC, CD166-PE (Biolegend); HLA-DR (eBioscience). HLA-G immunofluorescence was performed using standard methods. Briefly, cells seeded on chamber slides were fixed with 4% paraformaldehyde, permeabilized with 0.2% triton X, blocked with 5% goat serum and incubated with 1:100 (5 μg/ml) anti-denatured HLA-G antibody, clone 4H84 (BD Biosciences) or 1:200 (5 μg/ml) mouse IgG1 isotype control overnight. The next day, a 1:200 dilution of Cy3-labelled goat anti-mouse secondary antibody (Jackson Labs) was applied followed by a 2 μg/ml solution of DAPI to stain nuclei. Cover slips were attached with Vectashield hard mount. Images were acquired with a 20X UplanFluor objective (NA = 0.45) on a Nikon Eclipse TE2000-S microscope and analyzed with QCapture software (QImaging, Surrey BC Canada).

### NZB x NZW F1 Lupus Mouse Model: hESC-MSC administration

Female NZBxNZW F1 (BWF1) mice were obtained from Jackson Laboratories (Bar Harbor, Maine) and housed at Tufts University Veterinary Center (Grafton, MA). These mice were administered passage 4 hESC-MSCs 3 times weekly, or 6 times bi-weekly, beginning at week 23–24, as indicated. hESC-MSCs were administered intravenously by tail vein injection at 5 × 10^4^ or 5 × 10^5^ cells per mouse in PBS or a glucose/saline solution, with control mice receiving injections of vehicle alone. All experimental animal protocols were approved by the Tufts University Institutional Animal Care and Use Committee and methods were carried out in accordance with their approved guidelines.

Beginning at 20 weeks of age, body weight and proteinuria were measured weekly to monitor disease progression. Week 20 served as the initial body weight baseline to determine the percentage change in weight as the mice aged. The following system was used to score the severity of proteinuria, by testing urine on colorimetric Albustix (Siemens): 0 is trace; 1 is 30 mg/dL; 1.5 is 30–100 mg/dL; 2 is 100 mg/dL; 2.5 is 100–300 mg/dL; 3 is 300 mg/dL; 3.5 is 300–2000 mg/dL; 4 is 2000 mg/dL. Repeated weekly measurements with Albustix provide a general estimate of proteinuria levels over time even though a single measurement does not take into account differences in urine volume. Since the level of BWF1 proteinuria increases by orders of magnitude as mice develop disease, repeatedly weekly monitoring with a dipstick method such as Albustix has been used in many studies, e.g.,^7^,^45^,^46^. Moreover, any mouse displaying a proteinuria reading above 3 and >15% body weight loss was re-analyzed for proteinuria on the following day. Mice were sacrificed if they displayed 2 consecutive daily proteinuria readings above 3 and >15% body weight loss or were otherwise moribund. A subset of mice was euthanized at wk 35 for age-matched kidney analysis.

### Kidney Histology

Histopathology was performed by Mass Histology (Worcester, MA). Kidneys from mice at 35 weeks of age were fixed in 10% buffered formalin (Fisher Scientific), sectioned, and H&E/PAS-stained. Immunohistochemical staining was performed for CD3 infiltration (Thermo Scientific) on fixed kidney sections. Slides were scored by a blinded, board-certified veterinary pathologist, following a previously established scoring system for kidneys from lupus-prone mice^47^. In brief, glomeruli were scored from 0–6, with 0 being no observed lesions, 1–2 mild lesions with mesangial deposits and/or hypercellularity, 3–4 moderate to severe lesions with mesangioproliferation, wire-loop capillaries and/or fibrous necrosis of capillary loops, intraglomerular crescent formation, ruptured Bowman’s capsule and/or periglomerular inflammation affecting <25% of the glomeruli or 25–50% of the tufts, and 5–6 being severe disease characterized as in 3–4 but affecting more than 50% of the glomeruli or more than 75% of the tufts. Interstitial inflammation was scored from 0–4 for no (0), minimal (1), mild (2), moderate (3) or severe (4) inflammation and fibrosis. Intratubular protein cast formation was also scored 0–4, 0 was for no protein deposition in the renal tubules and scores of 1–4 were used when there was 5%, 5–25%, 25–50% or > 50% of renal tubules affected, respectively. IgG staining was performed on acetone-fixed cryosections from kidneys of 35 week-old mice with Alexa Fluor 488-labeled anti-mouse IgG from Abcam (ab150105).

### Serum Analysis

Serum from 35 wk old control and hESC-MSC-treated BWF1 mice was analyzed for creatinine and blood urea nitrogen (BUN) at the Yale Mouse Metabolic Phenotyping Center (New Haven, CT). Serum anti-ds DNA antibody levels were determined with a total Ig ELISA kit from Alpha Diagnostics International (San Antonio, TX) according to manufacturer’s instructions.

### *In vitro* Co-culture with hESC-MSCs

hESC-MSCs were plated in 24- or 48-well plates and allowed to attach overnight. The next day, bone marrow mononuclear cells (BMMC) from vehicle-treated 35 wk old BWF1 mice were added at a ratio of 10 to 1 (BMMC:hESC-MSC) in IMDM + 10% heat-inactivated Hyclone FBS. After 2 days, 10 ng/ml lipopolysaccharide (LPS, Sigma Aldrich, St. Louis, MO) was added to the co-cultures and incubated for an additional 3 days, at which time cells were harvested and subjected to flow cytometry for mouse CD4, CD25, and propidium iodide for viability. Supernatants were collected for CBA analysis.

### Cytokine Analysis

Cytokine analysis was performed on serum and supernatant from 24h LPS-stimulated BMMC +/− hESC-MSC co-culture using a Th1/Th2/Th17 Cytometric Bead Array (BD Biosciences) per manufacturer’s instructions followed by analysis on a BD Accuri C6 flow cytometer.

### Statistical Analysis

Two-tailed student’s t-test and the non-parametric Mann-Whitney test were used to determine p-values as indicated. For survival graphs, the log rank test was used.

## Additional Information

**How to cite this article**: Thiel, A. *et al*. Human embryonic stem cell-derived mesenchymal cells preserve kidney function and extend lifespan in NZB/W F1 mouse model of lupus nephritis. *Sci. Rep.* **5**, 17685; doi: 10.1038/srep17685 (2015).

## Supplementary Material

Supplementary Information

## Figures and Tables

**Table 1:** Proteinuria and kidney histopathology disease scores for individual mice.

**Treatment Group**	**Proteinuria** S**core**	**Glomeruli**	**Interstitium**	**Protein Cast**
Ctrl	1	0	1	0
Ctrl	4	5	3	4
Ctrl	4	4	2	3
Ctrl	3	6	3	3
Ctrl	1.5	1	1	0
Ctrl	3.5	6	1	3
Ctrl	4	6	2	3
Ctrl	1	1	2	1
Ctrl	0	1	2	0
hESC-MSC	1	0	1	0
hESC-MSC	1	0	0	0
hESC-MSC	0	1	1	0
hESC-MSC	0	0	0	0
hESC-MSC	0	0	0	0
hESC-MSC	0	1	1	0
hESC-MSC	0	0	1	0
hESC-MSC	1	1	1	0
hESC-MSC	3	2	2	0

*1 hESC-MSC-treated mouse (proteinuria score 4) was found dead in cage, unable to perform histopathology.
