# Supplementary material for: Human embryonic stem cell-derived mesenchymal cells preserve kidney function and extend lifespan in NZB/W F1 mouse model of lupus nephritis
Source: Sci Rep. 2015 Dec 2;5:17685. doi: 10.1038/srep17685 (PMC4667213; doi:10.1038/srep17685)
Supplement: Supplementary Information [file srep17685-s1.pdf]

Supplementary Information for:

Human embryonic stem cell-derived mesenchymal cells preserve kidney function and extend lifespan in NZB/W F1 mouse model of lupus nephritis

Austin Thiel, Gregory Yavanian, Maria-Dorothea Nastke, Peter Morales, Nicholas A. Kouris, Erin A. Kimbrel, and Robert Lanza

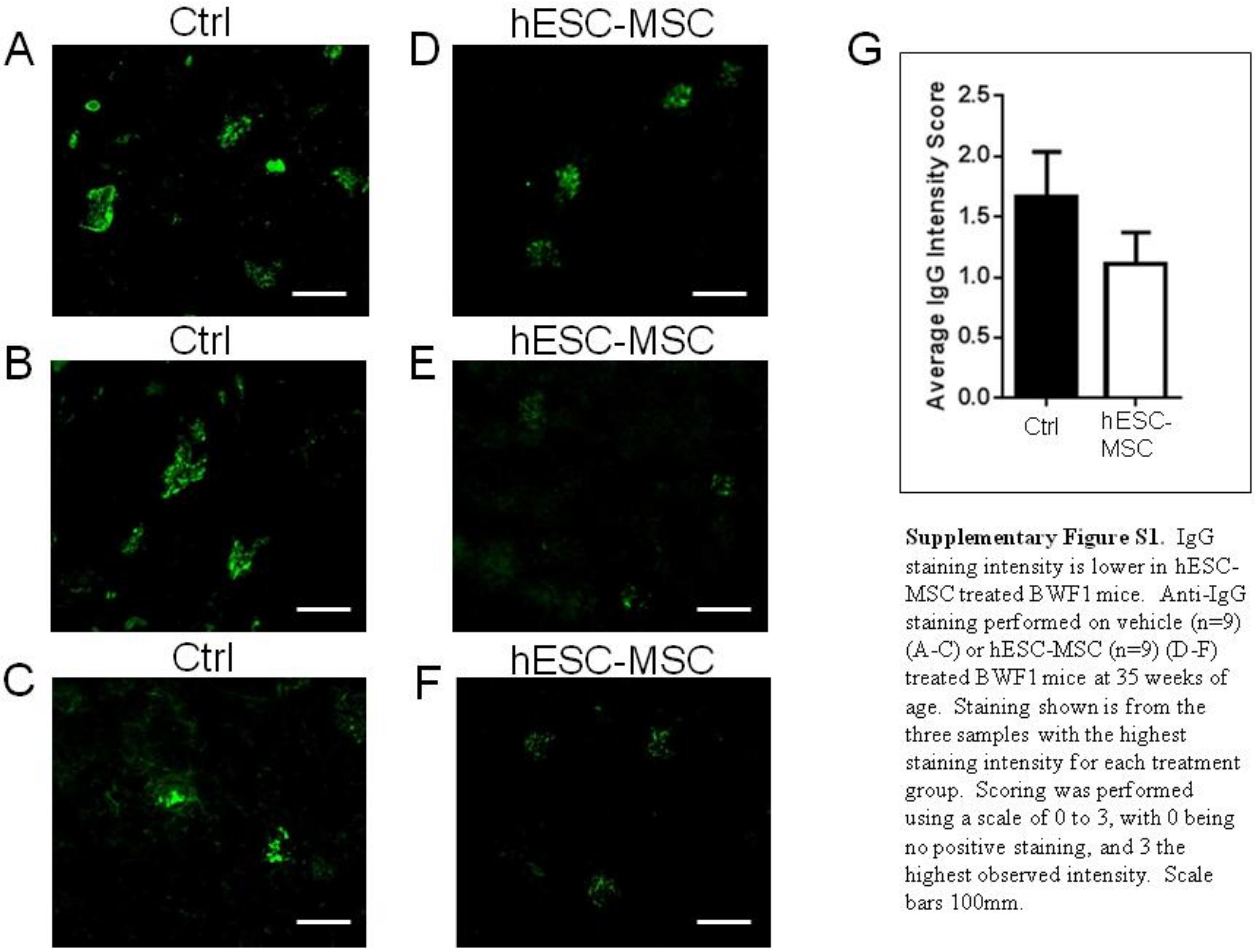

Supplementary Figure S1. IgG staining intensity is lower in hESC-MSC treated BWF1 mice. Anti-IgG staining performed on vehicle (n=9) (A-C) or hESC-MSC (n=9) (D-F) treated BWF1 mice at 35 weeks of age. Staining shown is from the three samples with the highest staining intensity for each treatment group. Scoring was performed using a scale of 0 to 3, with 0 being no positive staining, and 3 the highest observed intensity. Scale bars 100mm.

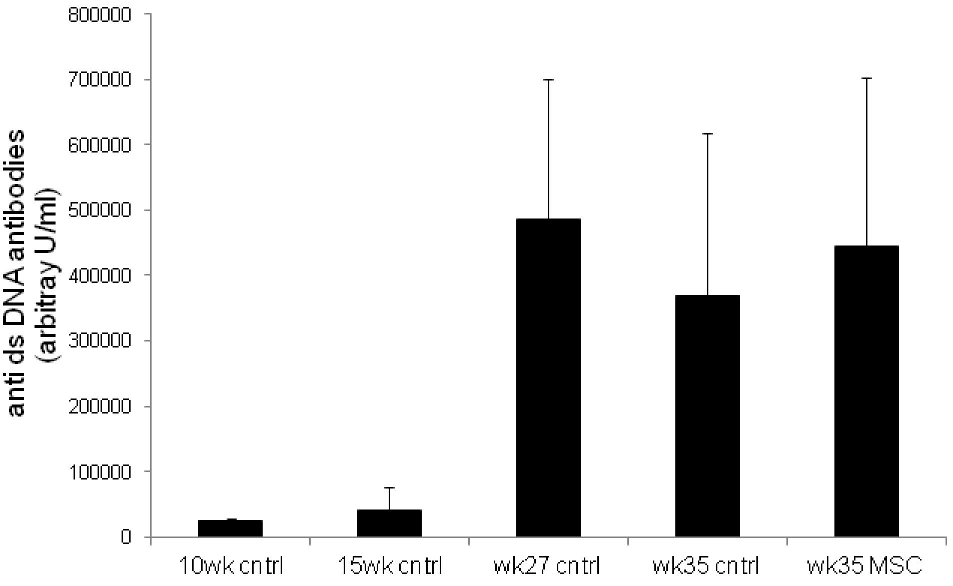

Supplementary Figure S2. Serum levels of anti ds DNA antibodies do not differ between untreated controls and hESC-MSC-treated BWF1 mice at 35 wks of age. Graph shows the level of anti ds DNA antibodies in different groups of untreated control BWF1 mice at 10 wks of age (n=5), 15 wks (n=5), 27 wks (n=13), 35 wks (n=12) of age versus the level in hESC-MSC-treated mice at 35 wks of age (n=11). Bars represent the average +/- SD for each group.
